# Cardiopulmonary exercise testing in pulmonary arterial hypertension and chronic thromboembolic pulmonary hypertension

**DOI:** 10.3389/fspor.2024.1477562

**Published:** 2024-11-20

**Authors:** Alexis Coulis, Shir Levanon, Gurshaun Randhawa, Yevgeniy Brailovsky, Farhan Raza, Estefania Oliveros

**Affiliations:** ^1^ Temple Heart and Vascular Institute, Temple University Hospital, Philadelphia, PA, United States; ^2^ Jefferson Heart Institute, Thomas Jefferson University, Philadelphia, PA, United States; ^3^ Department of Medicine-Cardiovascular Division, University of Wisconsin Hospital, Madison, WI, United States

**Keywords:** pulmonary hypertension, chronic thromboembolic pulmonary disease, chronic thromboembolic pulmonary hypertension, cardiopulmonary exercise testing, pulmonary embolism

## Abstract

Cardiopulmonary exercise testing allows for a comprehensive assessment of the mechanism of exercise limitation in pulmonary arterial hypertension and chronic thromboembolic pulmonary hypertension. Competitive pathophysiologic mechanisms may affect the clinical interpretation of cardiopulmonary disease as they relate to dyspnea, leg fatigue, and exercise intolerance.

## 1 Introduction

Cardiopulmonary exercise testing (CPET) is a diagnostic tool used to investigate functional capacity and characterize exercise limitations related to circulatory impairment and ventilatory inefficiency (1, 2). It is a durable and versatile test that provides invaluable information on a patient's cardiovascular and pulmonary disease. Traditionally the 6 min walk distance is routinely used to assess the functional capacity of patients with PH for risk stratification, but can lack cardiopulmonary specificity. Therefore current ESC/ERS guidelines (3) recommend CPET for further evaluation in symptomatic patients with intermediate echocardiographic findings of pulmonary hypertension (PH), therefore we will try to provide key parameters to understand and interpret those findings. CPET allows the analysis of gas exchange at rest as well as during exercise and recovery, using breath-by-breath analysis of oxygen uptake (V_O2_), carbon dioxide output (V_CO2_), and ventilation (V_E_). This data is integrated with measurements of symptoms, heart rate, blood pressure, work rate, and electrocardiographic findings.

The definition of PH was recently updated to emphasize early disease detection in the guidelines from the World Health Organization (WHO) -European Society of Cardiology (3). Currently, PH is defined as a mean pulmonary artery pressure (mPAP) of greater than 20 mmHg and pulmonary vascular resistance (PVR) of greater than 2 Wood Units. There are 5 clinical categories based on the etiology of PH, and we will focus on pulmonary arterial hypertension (PAH)- WHO Group 1, and chronic thromboembolic pulmonary hypertension (CTEPH)- WHO Group 4.

PAH is precapillary PH characterized by endothelial dysfunction leading to increased pulmonary vascular resistance (3). It can be idiopathic, hereditary, or associated with predisposing diseases. CTEPH is an uncommon complication of pulmonary embolism (PE) defined by the presence of precapillary PH with angiographic evidence (i.e., computed tomography of the chest, magnetic resonance imaging of the pulmonary arteries, catheter-based pulmonary angiography) of organized thrombotic residua within the pulmonary arteries that are present in spite of at least 3 months of anticoagulation therapy. For those with imaging evidence of chronic PE in the absence of rest-hemodynamic evidence of PH, the diagnosis of chronic thromboembolic disease (CTED), or most recently the proposed designation, chronic thromboembolic pulmonary disease (CTEPD), is suggested.

In both PAH and CTEPH, there are multiple variables affecting cardiac function and pulmonary gas exchange leading to exercise intolerance and exertional dyspnea. These changes reflect the underlying exercise pathophysiology and can be evaluated via CPET. This review summarizes the developments and evidence on the application of CPET in PH.

## 2 Practical considerations and key parameters of cardiopulmonary exercise testing

The usefulness of the CPET depends on an individual's capacity to perform physical exercise, evaluating the ability of the pulmonary system's ability to clear CO_2_ and the cardiovascular system's ability to supply oxygen to the muscles. There is a close evaluation of the delivery and removal systems (i.e., ventilation, diffusion, transport of O_2_ and CO_2_ in the blood, and capillary gas exchange). The increase in oxygen uptake by the peripheral muscles is a response to an increase in cardiac output (CO = heart rate × stroke volume), which increases 6 times with exercise. The cardiac output is distributed to skeletal muscles and shunted away from the non-active tissues (e.g., splanchnic circulation). There is an increase in blood flow to the lung in addition to vasodilation of the pulmonary vessel, with increased extraction of O_2_ from the blood to the muscles, resulting in an increased arteriovenous oxygen difference. Ventilation (V_E_) increases proportionally with the work rate. When inhalation takes place, the air in the alveoli, known as the tidal volume (Vt), accounts for the gas exchange. The air not participating in this exchange, is known as the dead space (Vd). During exercise, both dilation of the respiratory passages and the effective areas of gas exchange (tidal volume) increase, therefore gas exchange remains intact. The increase in V_E_ should be matched with an increase in blood flow, hence an appropriate cardiac output is important to maintain gas exchange. The point at which oxygen uptake is unable to increase adequately for peripheral oxygen demand marks the aerobic-anaerobic transition at which lactic acidosis will begin to occur. This is called the anaerobic threshold. If at any point there is a mismatch in this supply-demand chain there will be a clinical symptom.

The assessment of exercise capacity can be performed via a treadmill or stationary cycle ergometer. The selection of the exercise modality will depend on the patient and center's availability. Treadmills are preferred. Meanwhile, cycle ergometers are preferred in cases of gait and balance instability, severe obesity, orthopedic limitations, or when simultaneous right heart catheterization or echocardiogram is planned. The Bal and Ware, Naughton, and individualized ramp protocol involve increments in work rate per stage. They are recommended, as they allow to establish relationships between measured V_O2_ and work rate. Individuals should be expected to exercise for 8–12 min (4). Table 1 summarizes the CPET variables that are measured and calculated during the study (1, 3). Normal values may be affected in some individuals depending on their age, gender, volitional effort, and comorbidities.

**Table 1:** Cardiopulmonary exercise test variables.

Nomenclature	Definition	Normal values
Metabolic and cardiovascular		
Peak V_O2_	Aerobic capacity	≥85% based on predicted V_O2_ or ≥20 mL O_2_/min/kg (5)
		≥10 mL · kg−1 · min−1 if treated with β-blockade
		≥14 mL · kg−1 · min−1 if not treated with β-blockade
V_O2_ at AT	Oxygen consumption at the onset of metabolic acidosis	≥40%–80% predicted V_O2_ (5)
V_O2_/WR	Dependence on anaerobic metabolism due to cardiac output and impaired peripheral muscle oxygen use	≥10 mL/min/W
Peak HR	Chronotropic response to exercise	220-age (>85% predicted)
O_2_ pulse	(V_O2_/HR) Stroke volume recruitment and correlation between an increasing cardiac output on heart rate	≥80% (5)
Heart rate recovery	Onset of parasympathetic tone and withdrawal of sympathetic tone (Maximum HR - HR one minute post exercise)	≥18 beats/min in the first minute post exercise
OUES	Oxygen uptake efficiency slope: ability to increase V_O2_ per 10-fold rise in ventilation	In men, OUES (L/min) = [1,320−(26.7 × age) + (1,394 × body surface area)]/1,000. In women, OUES (L/min) = [1,175−(15.8 × age) + (841 × body surface area)]/1,000 (6, 7)
Ventilation and mechanics		
Peak V_E_	Maximal ventilation achieved during exercise	
Breathing reserve	(1−V˙e/MVV) *100 < 20% and abnormally reduced	15%–20% (or ≥11–15 L/min) (5)
Dynamic hyperinflation	Increase in air trapping during exercise secondary to a shorter expiratory time	Decrease in inspiratory capacity <140 mL (8)
Gas exchange		
V_E_/V_CO2_ slope	Ventilatory efficiency	<30
V_E_/V_O2_ at AT	Ventilatory equivalent of O_2_, which is lowest near AT and then increases once compensatory hyperventilation takes place	<30 (9)
P_ETCO2_	Efficacy of CO_2_ delivery to alveoli (PACO_2_-PaCO_2_)	>28 mmHg
Saturation of O_2_	Oxygen saturation	>95%
P (A-a)O_2_	Efficacy of O_2_ uptake	10–20 at rest, 15–30 at peak
Vd/Vt	Ratio of physiologic dead space to tidal volume	0.25–0.35 at rest (should decrease with exercise) (1)

## 3 Physiology of exercise in PAH and CTEPH

During exercise, peripheral muscles have increased oxygen demand that is achieved by peripheral vasodilation and increased oxygen extraction. When the normal individual exercises, the cardiac output should increase to match this oxygen delivery for the increasing demand of peripheral muscles (10). In the lungs increased tidal volume and pulmonary blood flow (achieved via vasodilation and pulmonary capillary recruitment) allows for a proportional increase in alveolar oxygen uptake. The maximal achievable oxygen uptake is labeled as the peak O_2_ (peak V_O2_) (5). In the alveoli, the carbon dioxide is cleared by a corresponding increase in minute ventilation (V_E_ = Vt × respiratory rate). The point at which oxygen uptake is unable to increase adequately for peripheral oxygen demand marks the aerobic-anaerobic transition at which lactic acidosis will begin to occur. This point is called the anaerobic threshold.

**Figure 1:**
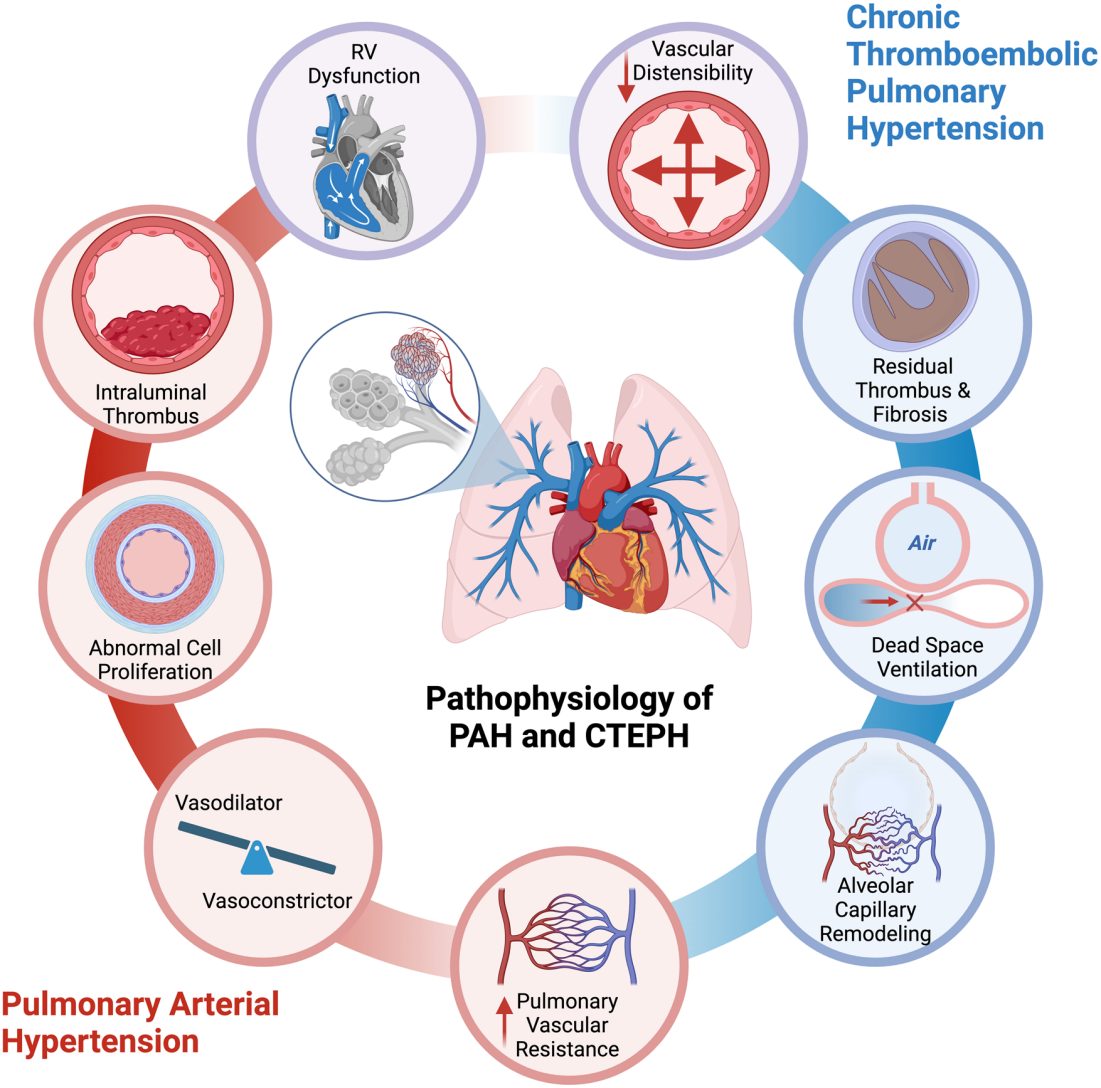
Pathogenesis of exercise limitation in pulmonary arterial hypertension and chronic thromboembolic pulmonary hypertension. (Created with biorender).

The physiological derangements in PH originate from obliteration and obstruction of pulmonary arteries (Figure 1). In PAH, vascular inflammation, remodeling, and endothelial dysfunction precipitate an increase in pulmonary vascular resistance. There is an imbalance in the production of pulmonary vasodilators and vasoconstrictors, abnormal proliferation of cells in the walls of the pulmonary arteries and arterioles, and intraluminal thrombus (11, 12). In CTEPH, residual thrombotic material causes pulmonary arterial obstruction, hindering pulmonary vasodilation and increasing dead space ventilation. In PAH and CTEPH, there is a decrease in the vasodilatory capacity, as well as in the distensibility and patency of the pulmonary vasculature. This hypoperfusion to ventilated lung regions is reflected as low P_ETCO2_. In both entities, PAH and CTEPH, there is an increase in afterload, or pulmonary vascular resistance, which limits the increased stroke volume needed for effective circulation at the time of exercise (10, 13, 14). The pulmonary vascular bed of PH patients is unable to be recruited and distended, leading to increased pulmonary artery pressures (10). The sustained increased afterload causes adaptive myocardial hypertrophy, which is initially reflected by RV wall hypertrophy, and later on by RV dilation (15, 16). There can be RV ischemia related to imbalanced oxygen supply and demand associated with hypertrophy, increased RV workload, and metabolic demands, without an increase in blood supply or microcirculation (17). In addition, there is uncoupling between the pulmonary vascular bed and the right ventricle, leading to right heart failure at peak exercise and inability to augment CO. In addition, elevated right ventricular pressure and dilation can cause interventricular septal shifting, which further reduces the pulmonary venous return to the left atrium, thereby decreasing left ventricular diastolic filling, systemic cardiac output, and tissue oxygen delivery (18–20).

The resulting hypoxia leads to vasoconstriction and impaired tissue oxygen delivery to peripheral muscles, causing lactic acidosis, lowered peak V_O2_, and a reduction in the anaerobic threshold. This leads to the production of V_CO2_ and an excessive increase in ventilation during exercise (increased V_E_/V_CO2_). This results in the characteristic CPET pattern of PH with low P_ETCO2_, high ventilatory equivalent for carbon dioxide (V_E_/V_CO2)_, and low peak O_2_ uptake (peak V_O2_).

### 3.1 Indications and contraindications for CPET

Patients with PH can have nonspecific symptoms, such as fatigue, dyspnea, and exercise intolerance. CPET can help identify the source of exercise intolerance, monitoring disease progression or treatment response, and aiding in prognosis. Ultimately, CPET allows for physiologic risk stratification. Table 2 includes the absolute and relative contraindications. CPET can provide reproducible cardiovascular-specific information as you can record heart rate, electrocardiogram, blood pressure, and gas exchange patterns. The primary measurements made are respiratory rate, end-tidal pO_2_, and pCO_2_. Other parameters, such as O_2_ pulse, and ventilatory equivalents for O_2_ and CO_2_ are also obtained. Individuals should reach an RER >1.05. There are some limitations with the interpretation of the data depending on the volitional effort of the individual, but the interpretation can be done in the context of the maximum HR and RER achieved. The patient does not require prior training to achieve better performance. CPET can rule out other factors affecting the ability to exercise, such as anemia, pulmonary mechanical pathology, or skeletal muscle myopathy. CPET in patients with PH is highly reproducible and provide robust endpoints to assess exercise capacity (21).

**Table 2:** Absolute and relative contraindications for cardiopulmonary exercise testing (adapted from ATS/ACCP statement on cardiopulmonary exercise testing) (1).

Absolute	Relative
Acute myocardial infarction (3–5 days)	Left main coronary stenosis or its equivalent
Unstable angina	Moderate stenotic valvular heart disease
Uncontrolled arrhythmias causing symptoms or hemodynamic compromise	Severe untreated arterial hypertension at rest or hemodynamic compromise (>200 mm Hg systolic, >120 mm Hg diastolic)
Syncope	Tachyarrhythmias or brady-arrhythmias
Active endocarditis	High-degree atrioventricular block
Acute myocarditis or pericarditis	Hypertrophic cardiomyopathy
Symptomatic severe aortic stenosis	Severe pulmonary hypertension
Uncontrolled heart failure	Advanced or complicated pregnancy
Acute pulmonary embolus or pulmonary infarction	Electrolyte abnormalities
Thrombosis of lower extremities	Orthopedic impairment that compromises exercise performance
Suspected dissecting aneurysm	
Uncontrolled asthma	
Pulmonary edema	
Room air desaturation at rest ⩽85%*	
Respiratory failure	
Acute non-cardiopulmonary disorder that may affect exercise performance or be aggravated by exercise (i.e., infection, renal failure, thyrotoxicosis)	
Mental impairment leading to inability to cooperate	

*multiplication sign.

Although CPET is generally well-tolerated, contraindications include physical barriers to exercise or decompensated disease state including but not limited to active myocardial ischemia, uncontrolled arrhythmia, severe aortic stenosis, COPD exacerbation, uncontrolled asthma, or other acute respiratory failure (22). Barriers for implementation can be related to confidence in interpreting results and lack of equipment or trained technicians.

### 3.2 Cardiopulmonary exercise testing in pulmonary arterial hypertension

**Figure 2:**
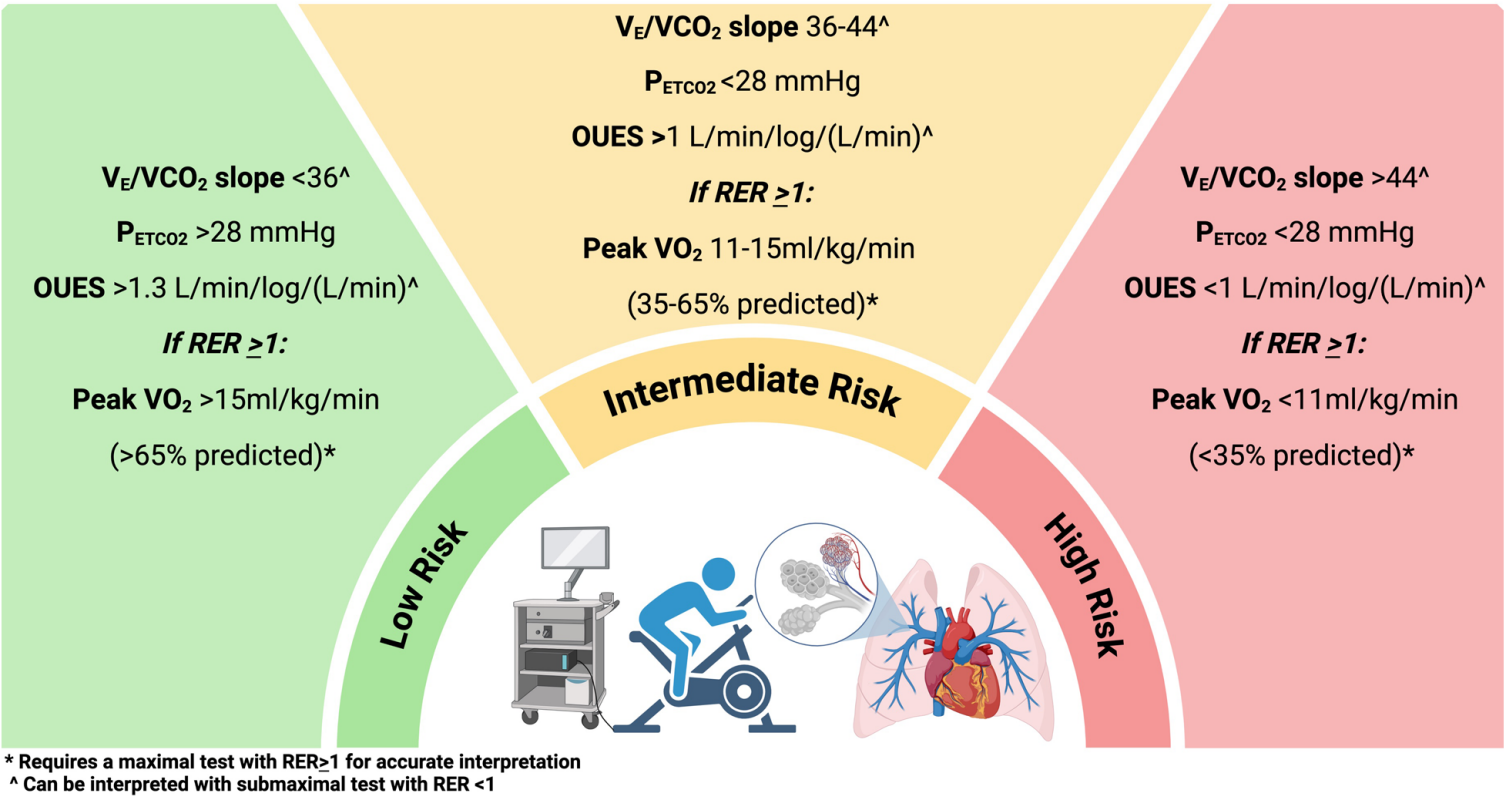
Cardiopulmonary exercise testing stratification in pulmonary arterial hypertension and chronic thromboembolic pulmonary hypertension. (Created with biorender).

The goal of CPET in PAH is to provide an early diagnosis, help stratify individuals with intermediate risk PH, identify peripheral muscle abnormalities related to PAH, and prognosticate and evaluate the efficacy of therapeutic interventions (23, 24) (Figure 2). The use of CPET in PAH was derived from its use in left heart failure (25). The mechanisms that impair exercise in PAH are: (1) inability to vasodilate the pulmonary vascular bed (i.e., impaired distensibility and vasodilation and smaller vessels) (26); (2) reduction in the RV contractility (19); (3) chronotropic impairment (27); (4) abnormal gas exchange and hypoxemia; (5) chemoreceptor activation; (6) endothelial dysfunction; and (7) skeletal muscle myopathy. All of these are measurable with CPET. The main abnormalities recognized in PAH are called ventilatory inefficiencies and are determined by an abnormally high ratio of ventilation to CO_2_ production (V_E_/VCO_2_), as well as a low end-tidal pCO_2_ (P_ETCO2_), and thus dead space ventilation, which in turn have proven to be reliable physiologic signatures of pulmonary vascular disease across a variety of PH conditions (28–31). Peak V_O2_ is prognostic in PAH. Wensel et al. (32) first reported the prognostic capability of CPET in PAH showing that a low peak V_O2_ of 10.4 mL/kg/min and a low peak exercise systolic blood pressure of less than 120 mmHg were associated with worse survival as compared to those with one or none of those factors.

Oxygen pulse reflects the capacity and efficiency of the heart to deliver oxygen per heartbeat and can be related to stroke volume or peripheral arterial extraction. Reduction in O_2_ pulse as the work rate increases suggests an issue with oxygen delivery. The ratio of change in oxygen consumption to changes in work rate (exercise factor) is usually 10 mL/min/W in healthy individuals. If this ratio cannot be achieved, it suggests impairments in the cardiac output with exercise. The gas exchange parameters will imply the presence of PH, but ultimately cardiac catheterization will be required to make the diagnosis of PAH.

With regards to diagnosis, current ESC/ERS guidelines include CPET among the recommended evaluations for patients with unexplained exertional dyspnea with indeterminate echo findings for PH (3). In symptomatic patients with systemic sclerosis, CPET with normal peak V_O2_ suggests against the presence of PH, reducing the need for screening RHC (33). Similarly, for patients with connective-tissue diseases, HIV, or portal hypertension, CPET is a recommended modality to screen for PAH. Additionally, CPET as an endpoint in clinical trials has been used in many consensus working groups.

### 3.3 Cardiopulmonary exercise testing in chronic thromboembolic pulmonary disease

Patients with persistent symptoms after an acute pulmonary embolism with a “post-PE syndrome” can benefit from further characterization of their impairment (34). Patients with CTEPH and CTEPD can have similar symptoms and perfusion defects, but the difference is in the lack of resting PH in the latter. Investigators have described in CTEPD a marked reduction in peak V_O2_, high V_E_/VCO_2_ at anaerobic threshold, and slightly decreased P_ETCO2_ (35). In a study combining CPET with right heart catheterization and stress echocardiography, Peak V_O2_ was negatively correlated with mPAP and PVR, while V_E_/VCO_2_ at AT was positively correlated with mPAP and PVR (35).

Regarding patients with concerns for CTEPH, CPET is included in the recommended workup along with echocardiography and BNP/NT-proBNP (36). This is corroborated by data from Held et al. (37), who described that a score combining P_(A−a)_O_2_, P_(c−ET)_CO_2_ (capillary to end tidal carbon dioxide gradient), P_ETCO2_ at anaerobic threshold, and V_E_/VCO_2_ slope, had a sensitivity of 83.3% and a specificity of 92.2% for the diagnosis of CTEPH, even in patients whose echocardiogram was normal. Zhu et al. (38) divided CTEPH patients by levels of severity defined by mean pulmonary artery criteria, and described the parameters associated with significant differences in oxygen uptake efficiency slope and plateau, V_E_/V_CO2_, peak V_O2_ and V_E_ at anaerobic threshold depending of the pulmonary artery pressure.

CPET is also useful to monitor response to treatment and assess disease severity in CTEPH. It has been used to monitor response in surgical patients post pulmonary thromboendarterectomy, balloon pulmonary angioplasty and inoperable patients medically managed with PDE5 inhibitors or endothelin receptor agonists (39, 40).

The differences between PAH and CTEPH have been described by some authors. Sun et al. (41) have described lower P_ETCO2_ than in PAH when compared to CTEPH patients. Other parameters that have been compared is the response of oxygen uptake efficiency at peak and anaerobic threshold, and patient with PAH have higher values than patients with CTEPH not in proportion with hemodynamic severity (42).

## 4 Conclusion

As PAH and CTEPH diagnosis and management continue to improve the evaluation of the exercise capacity of individuals with the use of CPET may be a key tool to understand the true source of their limitations. Abnormal responses to exercise can be better characterized with the use of CPET and allow for physiologically tailored interventions. We can help stratify the disease via this comprehensive and reproducible method.
